# Morphometric Analysis of the Dimension and Distance to Anatomical Structures From the Mental Foramen Using Cone Beam Computed Tomography

**DOI:** 10.7759/cureus.71496

**Published:** 2024-10-14

**Authors:** Beenish Fatima Alam, Asilah Yusof, Shahzad Ali Shah, Johari Yap Abdullah, Mohamad Arif Awang Nawi

**Affiliations:** 1 School of Dental Sciences, Universiti Sains Malaysia, Kubang Kerian, MYS; 2 Oral Biology, Bahria University Medical and Dental College, Karachi, PAK; 3 Conservative Dentistry and Endodontics, College of Dentistry, Qassim University, Ar Rass, SAU; 4 Dental Research Unit, Center for Transdisciplinary Research (CFTR), Saveetha Dental College, Saveetha Institute of Medical and Technical Sciences, Saveetha University, Chennai, IND; 5 Biostatistics Unit, School of Dental Sciences, Universiti Sains Malaysia, Kubang Kerian, MYS

**Keywords:** alveolar crest, anterior mandible, diameter, inferior cortex of the mandible, pakistan

## Abstract

Objectives: This study aimed to determine the dimensions and differences in distances from several anatomical structures to the mental foramen (MtF) in Pakistani participants using cone beam computed tomography (CBCT).

Methods: For this cross-sectional study, retrospective CBCT data of Pakistani individuals from both genders were assessed using the Mimics software (Materialise NV, Leuven, Belgium). The participants were selected from the Mahajir and Pukhtoon ethnic groups in Pakistan. The dimensions of the MtF, which included vertical and horizontal diameter and area of foramen, were measured. The distance of the foramen to various anatomical structures was measured, which included the alveolar crest, inferior border of the mandible, and anterior mandible. Data were assessed using SPSS version 28 (IBM Corp., Armonk, NY). Statistical analysis was performed using an independent sample t-test and a paired t-test. P-values greater than 0.05 and 0.001 were considered significant.

Results: Greater measurements had been recorded for the Pukhtoon ethnicity with respect to the vertical, horizontal, and area of the foramen. In relation to the dimensions, males showed larger measurements than females. MtF's distance to the alveolar crest, inferior border of the mandible, and anterior mandible (p < 0.001) was greater in Pukhtoon ethnicity. Males displayed longer measurements.

Conclusion: CBCT proved to be a highly accurate and useful tool for the analysis of the dimensions and distances from the MtF in both ethnicities. The Pukhtoon ethnicity exhibited overall greater measurements with respect to the dimensions, highlighting a significant difference between the two ethnicities. Analysis of MtF distance to various landmarks resulted in longer measurements being observed in males and Pukhtoon ethnicity.

## Introduction

The mental foramen (MtF) is an opening situated on the anterior aspect of the mandible [1]. MtF provides a pathway for the mental nerve, which is a subdivision of the inferior alveolar nerve. This nerve provides innervation and sensory stimuli to the labial mucosa, lower lip, mandibular canines, and premolars [2]. The mental artery, which is a branch of the inferior alveolar artery, also passes through MtF and provides blood supply to the lower lip, surrounding mucosa, and chin [3]. The MtF can present as a single opening on each side of the mandible; occasionally, when more than one opening exists, these additional openings have been referred to as accessory mental foramina [4]. MtF has been observed to be present in the middle of the upper and lower border of the mandible. Additionally, it more commonly lies between the root apices of the mandibular first and second premolars [3]. However, the position of the MtF has been stated to differ in different ethnicities [5]. In the study by Phillips et al., MtF was mostly found at the mesial aspect and underneath the root apex of the mandibular second premolar [6].

Anesthesia of the mental nerve is commonly performed before planning out various surgical procedures, which include extractions, root canal procedures, placement of dental implants, periapical treatments, and treatment of traumatic injuries. Hence, the treating surgeons need to have accurate knowledge regarding the anatomical structures in close vicinity to avoid any complications [7]. The mental nerve may get affected or injured due to nerve compression as a result of inadequate infiltration of anesthesia into the foramen or due to neurotoxicity [8]. Hence, identifying the exact location of MtF is crucial to avoid unforeseen events.

Various techniques have been used in the past to evaluate the position, shape, dimensions, and distance to various anatomical landmarks. These involve using dry skulls, whereas the radiological analyses comprise periapical radiographs and panoramic radiography. These traditional two-dimensional imaging modalities collapsed a three-dimensional (3D) image into a two-dimensional image, which impacted the interpretation of the image as there was superimposition of anatomical structures. Consequently, it led to errors, along with magnification issues [9]. The advent of 3D imaging, such as cone beam computed tomography (CBCT), and computed tomography (CT), has overcome the issues faced using two-dimensional imaging. CBCT provides accurate 3D images of the anatomical structures within a short span of time and has lower radiation in comparison to CT scans [10].

CBCT has a cone-shaped X-ray beam placed in the middle of the detector; it generates high-quality, multiple-dimensional images without any distortions. It is quite efficient in generating images in about five to 40 seconds. CBCT generates a 3D view of the dental hard and soft tissues. These images could be visualized in axial, coronal, and sagittal views and could be further transformed into 3D images, which could be easily moved or rotated in different directions [10].

Several studies in the past have evaluated the dimensions and the position of the MtF [11,12]. Similarly, a study by Nanayakkara et al. measured the vertical diameter and reported it to range from 1.72 to 3.45 mm using the dried mandibles [3]. According to prior research by Laher et al., MtF was observed to be situated in the middle of the inferior aspect of the mandible and alveolar crest [7]. Most of the prior studies had been conducted on different races and many had used dried mandibles for assessment. Likewise, a previously conducted study in Pakistan by Shah et al. evaluated the number, location, and distance to the lower aspect of the mandible using CBCT only [13]. However, none of the previously conducted studies in Pakistan had measured the dimension and distance from MtF to various anatomical landmarks within the different ethnic groups. This study will be beneficial in helping surgeons recognize the ethnic variations that occur with respect to the anatomy of the MtF, which is crucial for improving surgical precision and minimizing adverse effects, as anatomical differences could impact surgical outcomes and the quality of patient care. This knowledge will be beneficial to the therapeutic planning of patients undergoing orthodontic treatment and maxillofacial and orthognathic surgery, during the placement of implants, and local anesthesia. Hence, this research aimed to determine the differences with respect to dimensions and distances from anatomical structures to MtF between the two ethnic groups from Pakistan, which are the Mahajir and Pukhtoon ethnicities using CBCT.

## Materials and methods

Study type and ethical approval

Data for this cross-sectional study were collected retrospectively. For the analysis, participants in the age group of 25-60 years, both male and female individuals from Pakistan, were assessed. Ethical approval for the study was obtained from Universiti Sains Malaysia prior to the commencement of the study.

Inclusion and exclusion criteria

Data of adult individuals from Pakistan belonging to Mahajir and Pukhtoon ethnicities from both genders were included. Data of individual's demographic details, which included their name, age, gender, address, telephone number, and ethnic details, were considered. Whereas, blurred or distorted images due to either participant movement or other issues, images with the presence of any abnormality, orthodontics, or traumatic injury in close approximation to the MtF, were not considered. Lastly, images in which patients' demographic details were not present were not considered.

Research groups

Two ethnicities from Pakistan were selected. These included Mahajir and Pukhtoon ethnicities due to differences in the overall appearance of these ethnicities. Mahajir group are residents of Sindh province, most of these people migrated from India in 1947 and have settled in Karachi, Pakistan. The Pukhtoon ethnic group belongs to people residing in the Khyber Pukhtoon Khuwa province of Pakistan. Some of these people share some genetic variation with people from Afghanistan.

Data collection & sampling technique

Retrospective data were collected from Pakistan. Author BFA was involved in collecting and analyzing the data. The data after being collected were then saved on the hard drive. The data were further visualized and assessed using the Mimics software (Materialise NV, Leuven, Belgium) on a computer. Name, age, gender, ethnicity, and addresses were used to confirm the ethnic group of the study participants. In case of confusion, telephonic calls were made to confirm the ethnicity of the participants. All the participants were segregated into two different groups based on their ethnicities and further divided based on their genders. To remove bias, all the demographic details of the participants from each ethnicity were concealed by the author (AY). To ensure no bias existed, authors AY and JYA re-examined the readings, following which they had been included in the study. Interexaminer reliability was found to be statistically significant (p < 0.001). A simple random sampling technique was used to collect the data.

Measurement procedure

Dimensions of MtF

**Figure 1:**
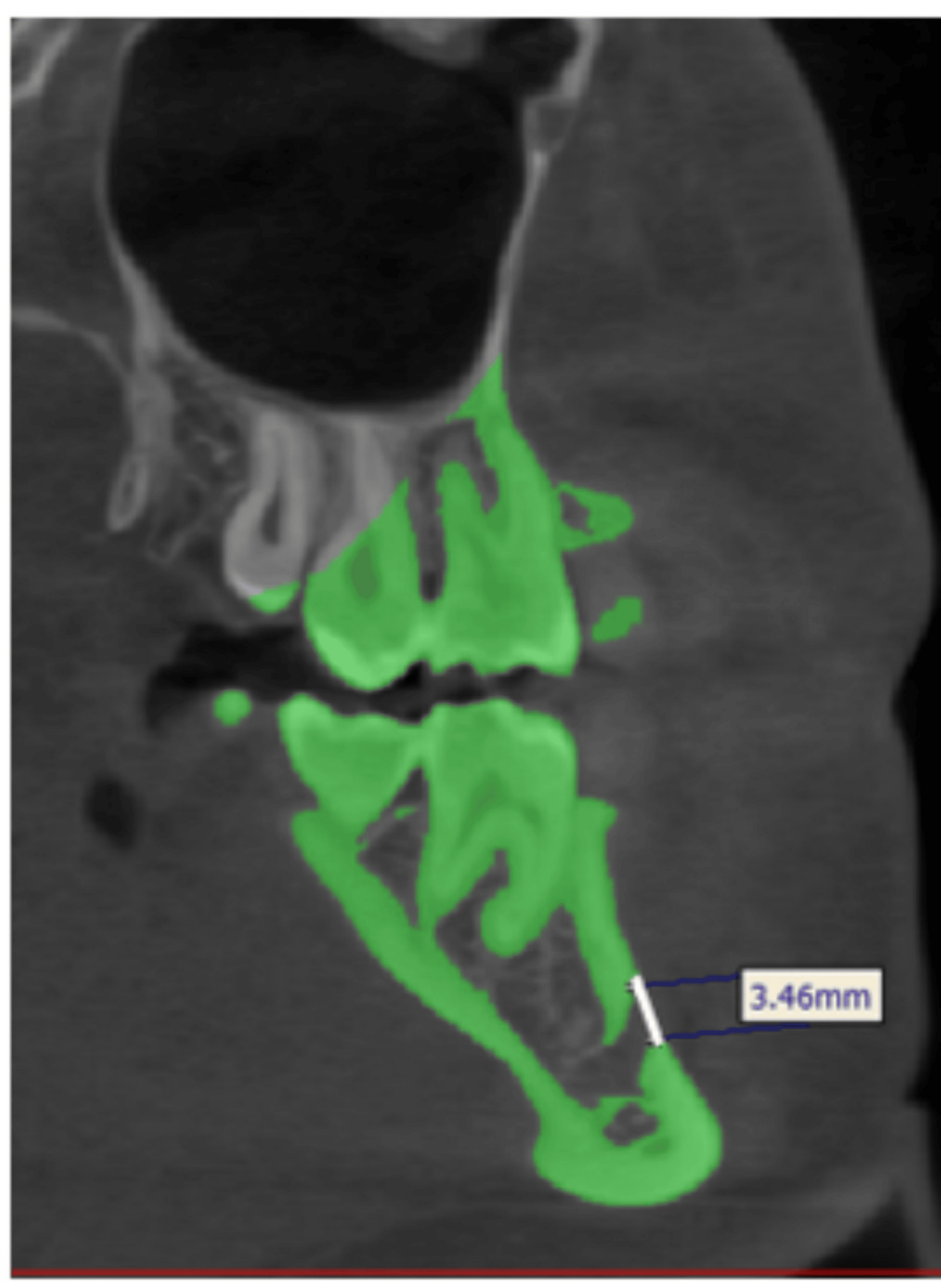
Vertical diameter of the mental foramen.

The vertical, horizontal, and overall area of the foramen were assessed. The vertical diameter was measured from the topmost margin to the lowermost edge of the foramen using the sagittal view (Figure 1).

**Figure 2:**
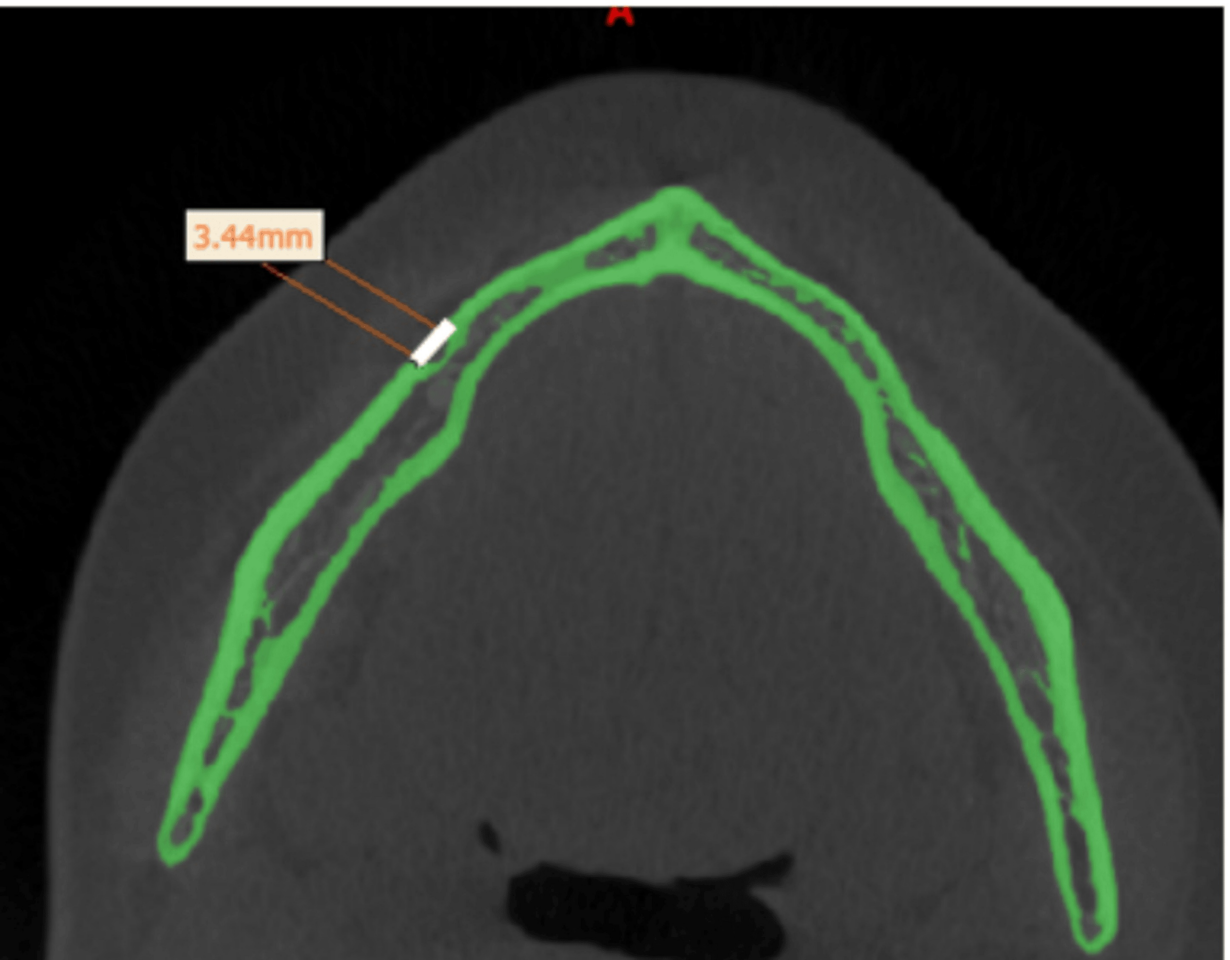
Horizontal diameter of the mental foramen.

**Figure 3:**
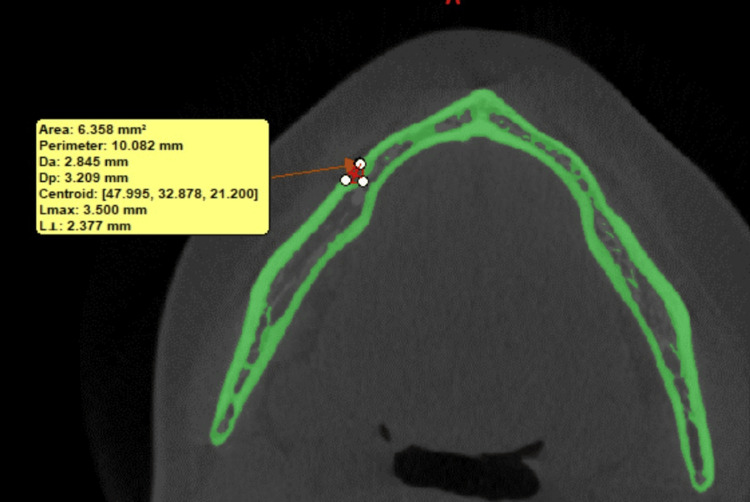
Measurement of the area of the mental foramen.

The horizontal diameter evaluated the mesiodistal width of the foramen while the area of the MtF was automatically calculated using the Mimics software, in which the area of interest is traced by placing small dots, which ultimately forms a small bounded region and for which the area is calculated by the software (Figures 2, 3).

Distance From MtF to Various Structures

**Figure 4:**
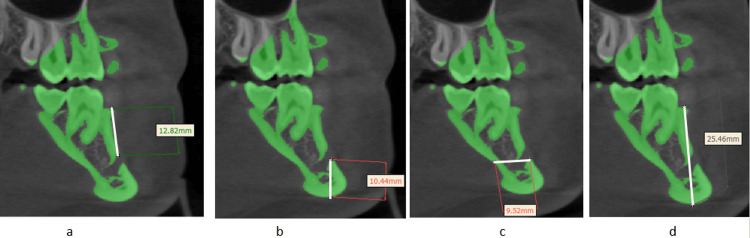
Measurements of distance from the MtF. (a) MtF to the alveolar crest. (b) MtF to the inferior border of the mandible. (c) MtF to the anterior border of the mandible. (d) Alveolar ridge to the inferior border of the mandible. MtF: mental foramen.

Distance from MtF to the alveolar crest was evaluated by measuring from the higher-most edge of MtF to the highest point on the alveolar crest. The second distance from MtF to the inferior part of the mandible was assessed from the lowermost part of MtF to the lowest edge of the inferior part of the mandible. The third distance from MtF to the anterior edge of the mandible was calculated by measuring from the inferior border of MtF to the anterior part of the mandible. Lastly, the distance from the alveolar ridge to the inferior border of the mandible was measured from the uppermost point of the alveolar crest to the bottommost point on the inferior part of the mandible (Figure 4).

Research tool

For this research, CBCT imaging was performed using the NewTom VGi (NewTom Co., Ltd., Shawnee, Italy). The positioning of the mandibular plane was fixed horizontally. The other scanning parameters that had been followed included the following: tube voltage = 110 kV; tube current = 0.583-1.05 mA; scanning window size = 12 cm x 8 cm; scanning time = 18 seconds; and voxel size = 0.15 mm.

The voxel size used was 0.08 mm while the field of view (FOV) was noted to be 10 x 6 cm. All the images for CBCT were obtained using 4.0 mA and 90 kV as the operating settings, and 5.6 seconds was the duration of exposure. All the generated CBCT images from the data were obtained in three dimensions (axial, sagittal, and coronal) and were reconstructed to obtain three-dimensional (3D) images.

For evaluating the CBCT images, Mimics software was used. It denotes “Materialise Interactive Medical Image Control System version 21 software.” This imaging software helps in processing 2D images that had been generated from CBCT to transform into 3D designs, 3D surface models, and 3D measurements. Additionally, this software generates images in different views, which include coronal, axial, and sagittal, and could further transform images into 3D views.

Sample calculation

The sample size was computed using the formula stated by Krejcie & Morgan [14]. This was the preferred method for the calculation of the sample during the evaluation of a large sample from a population. The mathematical link between the population size, confidence level, and margin of error yielded this formula. Because of this, this sample size is identified to be a valid and trustworthy technique for estimating sample size. The required sample was calculated to be 400. A total of 200 Mahajir and 200 Pukhtoon ethnic individuals were selected. Each ethnic group further comprised of 100 males and 100 females.

Statistical analysis

Statistical analysis was performed using SPPS version 28.0 (IBM Corp., Armonk, NY). To measure the dimensions of the MtF, an independent sample t-test was used. To measure the differences in the dimensions of the MtF, a paired t-test was performed. To assess the distance to different landmarks from MtF, an independent sample t-test was performed and to assess the difference between the sides, the paired t-test was used. P-values less than 0.05 and 0.001 were considered significant.

## Results

From a total of 400 participants, there were 200 individuals from the Pukhtoon ethnicity and 200 from the Mahajir group. Each ethnicity contained 100 males and 100 females. The age group of participants varied from 25 to 60 years.

Table 1 revealed the differences in the dimension of MtF between the two ethnic groups on both the right and left sides. In Mahajir ethnicity, the majority of the dimensions, with the exception of vertical diameter on both sides, showed insignificant findings (p = 0.376 and 0.194). Mahajir males exhibited greater dimensions in contrast to females in all of the measurements, regardless of the fact that few were not statistically significant. In Pukhtoon ethnicity, all measurements, excluding the left side vertical (p = 0.564) and horizontal diameter (p = 0.12), did not show significant differences. Pukhtoon males similarly displayed higher dimensions, though they were significant in most of the dimensions when equated to females (Table 1).

**Table 1:** The dimensions of MtF in two ethnic groups. * Significant at p < 0.05; ** significant at p < 0.001; independent t-test. MtF: mental foramen; RMtF: right mental foramen; LMtF: left mental foramen.

Dimensions & side	Gender	Mahajir				Pukhtoon			
		N	Mean (SD)	t (df)	p	N	Mean (SD)	t (df)	p
RMtF vertical diameter (mm)	Males	100	2.96 (0.953)	0.887 (198)	0.376	100	4.49 (1.145)	3.506 (198)	<0.001**
	Female	100	2.83 (1.072)			100	3.99 (0.849)		
RMtF horizontal diameter (mm)	Males	100	2.59 (0.789)	2.832 (198)	0.005**	100	3.29 (0.983)	3.487 (198)	<0.001**
	Female	100	2.30 (0.692)			100	2.87 (0.727)		
RMtF area (mm^2^)	Males	100	5.25 (1.639)	2.068 (198)	0.040*	100	5.63 (1.561)	2.277 (198)	0.024*
	Female	100	4.78 (1.650)			100	5.12 (1.594)		
LMtF vertical diameter (mm)	Males	100	3.11 (0.926)	1.303 (198)	0.194	100	3.40 (1.263)	0.577 (198)	0.564
	Female	100	2.93 (1.108)			100	3.30 (0.971)		
LMtF horizontal diameter (mm)	Males	100	2.60 (0.760)	4.560 (198)	<0.001**	100	2.87 (1.191)	1.561 (198)	0.12
	Female	100	2.16 (0.580)			100	2.63 (0.990)		
LMtF area (mm^2^)	Males	100	5.72 (1.751)	3.366 (198)	<0.001**	100	6.02 (2.056)	2.349 (198)	0.020*
	Female	100	4.88 (1.752)			100	5.37 (1.893)		

Table 2 shows the variances in the measurement of MtF on both sides and between the two ethnicities. In the Mahajir group, a statistically significant difference was observed with respect to the horizontal diameter and area. These dimensions were higher on the left side. In the case of Pukhtoon ethnicity, all dimensions demonstrated significant differences on both the right and left sides, while the left side showed greater measurements (Table 2).

**Table 2:** MtF dimension with respect to the side in ethnicities. * Significant at p < 0.05; ** significant at p < 0.001; paired sample t-test. MtF: mental foramen.

Dimensions	Side	Mahajir					Pukhtoon			
		N	Mean (SD)	t (df)	p	N		Mean (SD)	t (df)	p
Vertical diameter (mm)	Right	200	2.89 (1.013)	1.687 (199)	0.093	200		4.24 (1.036)	8.828 (199)	<0.001**
	Left	200	3.02 (1.023)			200		3.35 (1.124)		
Horizontal diameter (mm)	Right	200	2.44 (0.755)	7.229 (199)	<0.001**	200		3.08 (0.888)	3.046 (199)	0.003**
	Left	200	3.02 (1.023)			200		3.35 (1.125)		
Area (mm^2^)	Right	200	5.01 (1.657)	2.177 (199)	0.031*	200		5.37 (1.594)	2.304 (199)	0.022*
	Left	200	5.30 (1.796)			200		5.69 (1.998)		

Table 3 demonstrates the distance from MtF to various landmarks in both genders. The dimensions on the left side were greater for the distances to the alveolar crest, alveolar crest to the inferior part of the mandible, inferior part of the mandible, and anterior aspect of the mandible. Females revealed significant differences with respect to the alveolar crest and the inferior part of the mandible. Furthermore, longer distances were observed for the alveolar crest, inferior part of the mandible, alveolar crest to the inferior part of the mandible, and anterior aspect of the mandible on the left side (Table 3).

**Table 3:** Distances from MtF to various landmarks. * Significant at p < 0.05; ** significant at p < 0.001; paired sample t-test for differences. MtF: mental foramen; AC: alveolar crest, InfM: inferior part of the mandible; A to B: alveolar crest to the inferior part of the mandible; AntM: anterior aspect of the mandible.

Variable	Side	Male				Female			
		N	Mean (SD)	t (df)	p	N	Mean (SD)	t (df)	p
AC (mm)	Right	200	12.78 (2.400)	-2.438 (199)	0.016*	200	12.17 (1.990)	-7.710 (199)	<0.001**
	Left	200	13.19 (2.456)			200	13.53 (2.257)		
InfM (mm)	Right	200	13.51 (1.732)	-1.165 (199)	0.246	200	12.50 (1.503)	-16.241 (199)	<0.001**
	Left	200	13.77 (2.324)			200	14.26 (1.943)		
A to B (mm)	Right	200	28.49 (3.687)	-1.205 (199)	0.229	200	26.83 (2.853)	-1.472 (199)	0.142
	Left	200	28.75 (3.742)			200	27.13 (2.702)		
AntM (mm)	Right	200	14.84 (2.512)	-0.277 (199)	0.782	200	14.34 (2.163)	-5.413 (199)	0.123
	Left	200	14.89 (2.756)			200	15.43 (2.574)		

Table 4 denotes the MtF distance to landmarks on the right and left side between the ethnic groups. It was noted that only distances to the alveolar crest and the inferior border of the mandible in the Mahajir group exhibited significant differences between the two sides, while other variables did not show any significant variance. Furthermore, measurements were higher on the left side in this group. In Pukhtoon ethnicity, distances to landmarks, including the alveolar crest and the inferior and anterior border of the mandible, exhibited significant differences, with higher measurements noted on the left side (Table 4).

**Table 4:** MtF distances to landmarks between the two ethnicities. * Significant at p < 0.05; ** significant at p < 0.001; paired sample t-test for difference. AC: alveolar crest; InfM: inferior aspect of the mandible; A to B: alveolar crest to the inferior aspect of the mandible; AntM: anterior aspect of the mandible.

Landmarks	Side	Mahajir				Pukhtoon			
		N	Mean (SD)	t (df)	p	N	Mean (SD)	t (df)	p
AC (mm)	Right	200	11.78 (1.820)	-6.052 (199)	<0.001**	200	13.17 (2.373)	-4.187 (199)	<0.001**
	Left	200	12.77 (2.328)			200	13.94 (2.254)		
InfM (mm)	Right	200	12.92 (1.687)	6.052 (199)	<0.001**	200	13.09 (1.708)	7.980 (199)	<0.001**
	Left	200	14.23 (2.325)			200	14.81 (2.172)		
A to B (mm)	Right	200	27.25 (3.389)	1.450 (199)	0.149	200	28.07 (3.362)	1.237 (199)	0.218
	Left	200	27.54 (3.289)			200	28.34 (3.386)		
AntM (mm)	Right	200	14.84 (2.387)	0.461 (199)	0.646	200	14.34 (2.299)	4.806 (199)	<0.001**
	Left	200	14.92 (2.497)			200	15.40 (2.831)		

## Discussion

MtF is an anatomically significant landmark that needs to be considered before planning any surgical procedures, which includes placement of local anesthesia, incisions, placement of dental implants, conducting periapical procedures, and assisting osteotomies on the mandible [15]. The findings from the study revealed that Pukhtoon ethnicity displayed greater measurements with respect to the dimensions. Mahajir ethnicity displayed greater measurements on the left, whereas the Pukhtoon ethnicity had a larger diameter on the right side. According to a study by Budhiraja et al., nearly similar results had been observed, which were in line with our findings for the Mahajir group [16]. A Turkish study assessing dried mandibles showed that the average vertical diameter on the right was 2.93 mm, whereas on the left side, 3.14 mm was noted, which was in contrast to our findings [17]. This difference between the two sides could be because MtF is not symmetrical on both sides, and variation exists with respect to sides in races. In the current analysis, males displayed greater vertical diameters than females. These findings are consistent with an earlier analysis, which specified that diameter was greater in males and on the left side [12]. A Malaysian study reported marginally larger vertical dimensions in females [18]. Hence this identifies the fact that differences in size exist between different ethnicities and genders. These differences could be attributed to the type of investigation techniques used for analysis and the pattern of sexual dimorphism. Additionally, there are physical variations between the two ethnic groups. Mahajir people have short height and have a smaller physique as compared to the Pukhtoon ethnic group, who are mostly tall and have a larger build. This could be the cause for the foramens' larger overall sizes.

Overall, the horizontal diameter was comparatively higher in Pukhtoon ethnicity. An Indian study reported nearly similar findings, which were consistent with the outcomes of Pukhtoon ethnicity [19]. A Turkish study evaluated horizontal diameter using a dried cadaver and revealed that the mean size ranged from 2.93 to 3.14 mm, which was unlike our findings [17]. Our results were also in contrast with a study conducted on Malawian participants where the diameter ranged from 5.00 to 5.05 mm on both sides [20]. Horizontal diameter was greater in males in both ethnic groups. Studies conducted in Malaysia and India had shown similar findings, with males having a larger diameter [11,18]. MtF was greater in the Pukhtoon group on the left and in the Mahajir ethnic group on the right side. Few studies reported higher dimensions on the right side, which were consistent with our outcome regarding the Mahajir group [16,21]. It remains unclear why this dimorphism occurs. Sexual determination is an important aspect of forensic dentistry for the accurate identification of gender. The mandible, followed by the skull, are the most commonly used bones for this purpose. Moreover, it could also be connected to the male hormones. Further exploration is required. However, in our study, both vertical and horizontal diameters were larger in men, confirming the theory of sexual dimorphism.

The area of the MtF was observed to be greater in Pukhtoon ethnicity. According to Naitoh et al., the surface area of the MtF ranged from 3.4 to 19.7 mm, which was somewhat like our findings [22]. One scientific paper calculated the area of foramen by combining the long and short diameters of MtF using a formula. The area was found to be 11.71 mm in males and 9.80 mm in females, which differed significantly from our analysis [23]. These differences could be attributed to anatomical differences between racial and ethnic groups. Before initiating the treatment, the physician should understand the anatomy of the mental region. Therefore, having a clear 3D view of the jaw helps prevent any damage to this region, and minimizes the neurovascular disturbances. Moreover, the area was bigger in males, and on the left side. Spanish research also reported the area to be higher in men and stated comparable findings [23]. The area of the MtF has only been calculated by a few studies, and many have calculated it with the help of a formula. However, in the current study, Mimics software had been used to measure the area of the MtF. Mimics is a user-friendly software with features that help calculate the surface area of the foramen accurately.

The distance from the MtF to the alveolar crest was comparatively longer in Pukhtoon ethnicity. A prior study revealed a similar measurement to the alveolar crest [24]. Another study by Dos Santos Oliveira et al. also stated comparable distance, which ranged from 11.18 ± 1.99 mm to 11.29 ± 1.99 mm, which was consistent with this analysis [25]. Çaglayan et al. demonstrated comparable findings and reported this distance to be 11.19 to 13.10 mm [8]. Overall, this measurement varied between the races, genders, and ethnicities. Similarly, in existing analysis, variations were observed between the two ethnicities and gender, which might be due to the location of the foramen, and the level of bone present.

This measurement was greater in women on the left side while males had higher measurements on the right side. Few studies reported males having significantly longer measurements in comparison to females [16,26]. The study conducted by Asrani and Shah [26] measured this distance using panoramic radiography and reported it to be longer on the right side, which disagreed with our findings. Scientific investigation revealed that continual bone loss altered this distance [16]. The bone resorption as a consequence of tooth loss tends to push MtF near the alveolar crest, resulting in a lower bone height available for placement of dental implant. As a result of continued bone resorption, this results in shifting the foramen closer to the alveolar border. Additionally, during extensive resorption, the mental nerve and the remaining segment of an inferior alveolar nerve may lie in proximity to the alveolar edge or underneath the gums [27]. Henceforth, it is crucial that before performing any surgical practice in close vicinity to MtF, sufficient radiological examination must be performed to evade any risks.

The distance from MtF to the inferior border of the mandible was longer in the Pukhtoon ethnicity in comparison to the Mahajir group. The study by Sheikhi and Kheir reported a typical distance, which varied from 12.30 ± 2.13 mm to 14.38 ± 2.01 mm, which is in line with the current analysis [27]. Research by Neiva et al. assessed dried skulls and reported the average distance to be 10.33-13.67 mm, which is in accordance with the current analysis [28]. Findings by Dos Santos Oliveira et al. observed average distance to be 11.98 ± 1.58 in females and 13.13 ± 1.57 mm in males, which differed from our outcomes [25]. These differences between various studies could be due to different modalities used to compute this difference. These disparities could also be due to differences in the measurement point of MtF. In the current study distance from the midpoint of MtF was measured to the lower-most point of the inferior aspect of the mandible, whereas various previous studies had measured the distance of MtF either from its inferior or superior point, which could lead to differences.

Males had a longer distance to the inferior aspect of the mandible on the right side, while females exhibited longer measurements on the left side. Comparable findings had been stated by previously conducted studies, where males exhibited longer measurements in comparison to females [25,27]. Our outcomes complemented those of Chandra et al., who assessed this distance using panoramic radiographs and stated that males had longer distances [29]. This could be attributed to the fact that this distance does not fluctuate with age, making it a stable and consistent landmark, which could be used to verify gender. Concerning the difference between the two sides, this distance was longer on the left side. Similar results had been revealed in a previously conducted study, where distances were observed to be longer on the left side [27].

Distance from the alveolar ridge to the lower border of the mandible was higher in the Pukhtoon ethnicity. A previously conducted Malaysian study explored this measurement with the help of postmortem computed tomography and reported a slight difference in the measurements in the three ethnicities. In Malay ethnicity, the measurement was 2.54 ± 0.05 cm, in Chinese ethnicity, the measurement was 2.55 ± 0.05 cm, but in Indian ethnicity, it was 2.45 ± 0.05 cm, all of which contrasted with the outcomes of the current analysis [18]. Men in both groups displayed longer distances on the left side. Several studies reported similar outcomes where males exhibited longer distances [23,30]. However, Kabak et al. observed this distance to be longer on the right side, which was not consistent with the current study [30]. It was observed that the distance from the alveolar crest to the lower border of the mandible was equal to the distance from MtF to the alveolar ridge. This focuses on the impact of sexual dimorphism in this measurement.

The distance from MtF to the anterior aspect of the mandible was longer in the Mahajir ethnicity on the right side, whereas it was longer in the Pukhtoon ethnicity on the left side. Males displayed longer measurements. As none of the prior studies had assessed this distance from MtF to the anterior border of the mandible, we are unable to make a direct comparison. Our findings differed between the two ethnicities, which could be because MtF was situated away from the anterior border in Mahajir resulting in a longer distance on the right side, but it was closer on the left side, highlighting the pattern of asymmetry between the two sides.

Limitations

A few limitations of the study can be identified. Only two ethnic groups were considered for this analysis. The sample size for this analysis was 400; however, upcoming research could focus on increasing the sample size and adding participants from other ethnicities as well. Additionally, distance from MtF to other anatomical landmarks, which includes the coronoid process, condyle, ramus, and molars, was not considered but must be included in future studies. Lastly, operator blinding should have been conducted to remove the element of bias from the research.

The main strengths of the study included evaluating a sample of participants using CBCT, which provided an accurate analysis of various structures. Mimics software was used, which further aided in accurately analyzing and measuring the dimensions and distances to landmarks from the foramen. For future studies, data can be collected from different ethnicities of Pakistan, and measurements of other foramina, which include accessory mental, mandibular, and accessory mandibular foramen, could be performed using CBCT.

## Conclusions

CBCT, being a 3D imaging modality, was found useful in providing a highly accurate and precise analysis for all dimensions and distances to the MtF in both ethnicities from Pakistan. It was observed that Pukhtoon ethnicity exhibited overall greater measurements for most of the dimensions assessed for MtF, highlighting significant differences between the two ethnicities. Further examination of distance from MtF to various landmarks resulted in longer measurements being noted in males and Pukhtoon ethnicity.
